# Survey of Migratory Birds (Anatidae: *Anas platyrhynchos*) for *Schistosome* Parasites from Mazandaran Province, Northern Iran

**Published:** 2013

**Authors:** SA Mahdavi, A Farahnak, I Mobedi, MB Molaei Rad, H Azadeh

**Affiliations:** 1 Dept. of Parasitology and Mycology, School of Public Health, Tehran University of Medical Sciences, Tehran, Iran; 2 Prevention and Control of Disease Center, Mazandaran University of Medical Sciences, Sari, Iran

**Keywords:** *Trichobilharzia* sp., *Bilharziella* sp., *Anas platyrhynchos*, Iran

## Abstract

**Background:**

The aim of the present study was to survey birds’ schistosomes in migratory birds (Anatidae: *Anas platyrhynchos*) which are the source of the disease in Mazandaran Province, Northern Iran.

**Methods:**

A number of mallards were bought from the markets of hunted birds. The respiratory tracts (nasal mucosa) and intestinal blood vessels were studied for adult worms. The nasal mucosa was separated and observed by a microscope. In order to separate the visceral schistosomes, after separating intestine, vessel mesenteric was studied under the lamp light and then in saline. The parasite sample was collected for subsequent observation.

**Results:**

Fifteen (13.6%) cases out of 110 studied birds had nasal mucosa contaminated with *Trichobilharzia* sp. egg. Besides that, two birds had adult worms schistosome visceral i.e. *Bilharziella* sp.

**Conclusion:**

The elements that cause cercarial dermatitis in aforementioned region are *Trichobilharzia* sp. and *Bilharziella* sp. parasites. Thus, it is necessary for the authorities of health, environmental and agricultural organization of the province to cooperate in order to control this disease.

## Introduction

Skin lesion of cercarial dermatitis is red papules with skin irritation and itch, and finally after a while, blisters or scotches in the uncovered parts of hands and feet will appear due to the skin influx of cercariae of bird schistosomes, *Austrobilharzia, Bilharziella, Ornitobilharzia, Trichobilharzia, Gigantobilharzia*, and *dendrobilharzia* (1). Migratory aquatic birds specially mallards form Anatidae family are the final host (of adult warms in blood vessels) and the *Lymnae* mollusks are the intermediate host in the life cycle of this parasite (2). Thus, these migratory birds contaminate water, lay eggs in it, and contaminate mollusks. These mollusks release cercaria in water and cause illness in the people who are in contact with fresh waters (3).

This disease is one of zoonoses diseases and has been reported in the north (Gilan and Mazandaran Province) and south (Khuzestan Province) of Iran. On the other hand, this disease is economically and epidemiologically significant and is a new disease in Europe and a recurrent disease (due to migration of birds) in some parts of Iran (4–6).

It is mostly seen in rice farmers. If the farmers get this disease, they will be temporarily out of work. Since the climate of Mazandaran Province is good for the life cycle of this disease and different parts of this province are good places for the migratory birds to spend the winter, every year a considerable number of migratory birds which can contaminate the mollusks’ regional waters-and thus cause skin disease in the rice farmers- migrate to the northern province of Iran from the Central Asia.

The aim of the present work was to study birds’ schistosomes in mallards, which are the source of the disease.

## Materials and Methods

In this descriptive study began in December 2010, 110 birds (Anatidae; *Anas platyrhynchos*) were legally bought from the local hunters of Mazandaran cities, especially Ferydun Kenar. They were dissected in Sari Shahid Babaei Lab and Helminthology lab of the Department of Parasitology. The cold chain was maintained during the transference of these birds to laboratory. Since the present study focused on observing and studying the contamination of nasal vessels to egg and or adult helminthes family schistosomatidae, the birds’ head were dissected in the following way. First, the two parts of the break were opened and two deep cuts were made in the joint point of two jowls thus the two jowls completely opened. Then two linear cuts were made on the sides of the upper jowl and the palate bone and adjacent tissues were removed. In this condition, the nasal mucosa of both sides, which could be completely observed, had to be removed carefully for studying in lab. The birds’ heads were opened by a scalpel and the respiratory tract and the nasal mucosa were dissected and placed between two glass slides, pressed between them, and then was observed by a microscope and loop.

Some of the contaminated samples were put in alcohol of 70% and were transferred to the Parasitology Department of Tehran University for Further Studies. In addition, some samples were studied in the Helminthology lab of Parasitology Department and the positive samples were microscopically photographed in the central lab. For studying the respiratory tract and nasal mucosa just the egg helminths were separated and the suspicious slides were primarily put in alcohol of 70%, then after opening them and taking their tissue smear completely, these tissues were put in lactophenol and after transparent, were studies again. In addition to this, intestine mesenteric vessels of migratory birds were observed with loop after being washed by saline. The observed parasites were put on the glass slide by Pasteur pipette, another slide was put on them and then they were observed (5).

## Results

**Fig. 1:**
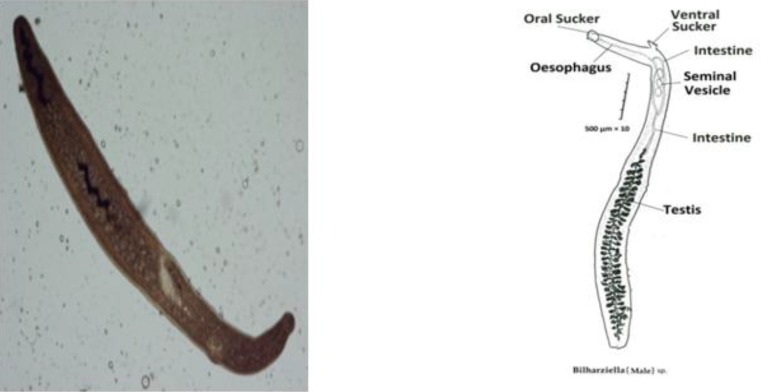
Male worm of *Bilharziella* sp. (Left: Photograph; Right: Drawing picture) (Original)

Totally 110 birds were studied. Sixty-three of these migratory birds were green head male dusks, ten of which (15.9%) had *Trichobilharzia* sp. egg. Forty-seven female ducks were studied and 5 (10.7%) had *Trichobilharzia* egg. Totally 15 cases (13.6%) were contaminated. Adult worms *Bilharziella* sp. were observed in two birds’ intestine mesenteric vessels (Fig. 1).

**Fig. 2:**
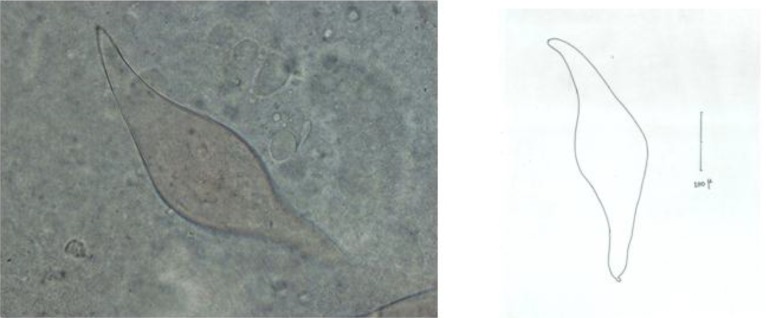
*Trichobilharzia* sp. egg. (Left: Photograph; Right: Drawing picture)

The noticeable characteristics of *Bilharziella* sp. are as follows:

body flat, large (up to 3-4 mm), but no filamentous; gynaecophoric canal absent; posterior end of body tapered and rounded; intestinal ceca unite near middle of body, common cecum without diverticula; suckers present and testes 68. The separated *Trichobilharzia* eggs are spindle-shaped, elongated, curved and sharp on both ends and have a jag on their end parts. These eggs are 253 micron long and 66 micron wide (Fig. 2).

## Discussion

Athari et al. (5) studied the prevalence of birds’ schistosomes in the final and intermediate hosts in Seyyed Mahale region of Sari. They reported that the contamination of the studied birds to *Trichobilharzia* was 18.1%. Besides, Shovele duck had the most contamination level and cercarial dermatitis was an important health issue in the region (5). Horak and Kolarova reported that the main element that causes birds’ schistosomes was genus *Trichobilharzia*. They also discussed the causes of cercarial penetration into human skin and its being limited to the skin and making dermatitis (1). Horak et al. investigated the pathological effects of birds’ schistosomes on lab animals and proposed theories about the dangers of cercaria penetration to skin for human, one of which was that cercaria probably involve other organs namely the lung tissue and spinal cord (7). Bayssade -Dufour et al. separated adult worm of *Bilharziella* from mesenteric vessels *A. platyrhynchus* and after studying the female and male gender and molecular investigation, they categorized it as *B. polonica* (8).

In the present study, migratory birds (ducks) had *Trichobilharzia* sp. egg but in two cases, the adult worm *Bilharziella* sp. was separated from mesenteric vessel. In a previous study, a population of 2310 people of Mazandaran Province was studied by same authors and it was found that 6% of them had cercarial dermatitis; this indicates the fact that parasite cycle exists in this province.

## Conclusion

Considering the prevalence of this disease and contamination of birds, it is necessary for the authorities of health, environmental and agricultural organization of the province to cooperate in order to control this disease.
